# Supplementary material for: Plasticity of muscle synergies through fractionation and merging during development and training of human runners
Source: Nat Commun. 2020 Aug 31;11:4356. doi: 10.1038/s41467-020-18210-4 (PMC7459346; doi:10.1038/s41467-020-18210-4)
Supplement: Supplementary file 1 — Supplementary Information [file 41467_2020_18210_MOESM1_ESM.pdf]

Supplementary Information for

Plasticity of Muscle Synergies through Fractionation and Merging during Development and Training of Human Runners

Vincent C. K. Cheung*†, Ben M. F. Cheung†, Janet H. Zhang, Zoe Y. S. Chan, Sophia C. W. Ha, Chao-Ying Chen, Roy T. H. Cheung*

Correspondence to: vckc@cuhk.edu.hk (Vincent C. K. Cheung) and Roy.Cheung@westernsydney.edu.au (Roy T. H. Cheung)

†Both authors contributed equally to this work.

This file includes:

Supplementary Notes 1 to 6
Supplementary Figures 1 to 7
Supplementary Tables 1 to 3
Supplementary Reference

SUPPLEMENTARY NOTES

Supplementary Note 1. Some elite muscle synergies could be explained by merging specific synergies from sedentary adults

To reveal the synergy merging patterns most related to improved running performance of the experienced runners, we directly compared the Sedent0 synergies with the Elite synergies. This comparison was achieved with two methods: (1) by matching the synergy-cluster centroids of the two groups after *k*-means clustering, and (2) by assessing the similarity between the synergy sets from each pair of subjects from the two groups (see Methods). With the first comparison method, we were able to match all 8 subject-invariant Elite clusters to Sedent0 clusters with high (scalar product, or SP ≥ 0.93; Supplementary Figure 1A, *E*-1 to 6) or moderate (SP = 0.81-0.87; *E*-7 to 8) similarity. In many of these matched pairs, however, the Elite synergy centroid tended to have active components over more muscles (i.e., less sparse) then its Sedent0 counterpart. This was especially obvious in the *E*-8/*S0*-8 pair (involving ankle, knee and hip extensors), but at least noticeable in all other pairs except *E*-4/*S0*-11 (Supplementary Figure 1A). The decreased sparseness of the Elite centroids, as compared with those of Sedent0, is consistent with the model that some Elite synergies are formed by merging multiple Sedent0 synergies.

We further characterized this merging of Sedent0 synergies by identifying all instances of synergy merging in all Sedent0-Elite subject pairs, and calculating the percentage of instances in which a synergy in any Sedent0 cluster contributed, through merging, to the explanation of a synergy in any Elite cluster (Supplementary Figure 1B). There were many instances of *S0*-5 (ankle extensors) and *S0*-6 (knee extensors) contributing to *E*-1, and *S0*-5, *S0*-6, and *S0*-8 (hip extensors) to *E*-8 (hip, knee and ankle extensors). To further clarify which Sedent0 synergies were merged in Elite, we listed all merging combinations that were observed in ≥1/3 of Elite (Supplementary Figure 1C). Of the 3 efficiency-enhancing merging combinations (colored text in Supplementary Figure 1C), 100% of the instances of 2 combinations (*S0*-5+6+8 and 5+6+12; E2 in Fig. 6) resulted in merged synergies in cluster *E*-8 whose centroid was the least well matched to Sedent0 (Supplementary Figure 1A). Thus, at least some of the differences between the Sedent0 and Elite clusters are attributable to the merging of the former in the latter.

Supplementary Note 2. Muscle synergies from sedentary adults were the most generalizable across the EMGs of different groups

Our demonstrations of how the Presch synergies were fractionated to give rise to the Sedent0 synergies and how the Sedent0 synergies could be merged to explain those in the more experienced runners (Fig. 2-3) imply that, among the synergy sets from the different groups, those in Sedent0 should span the largest volume of EMG space. The Sedent0 synergies should then also be the ones that are most generalizable in terms of being able to explain the variances of the EMGs of other groups. We therefore proceeded to systematically assess the across-group generalizability of the synergies from each group to validate the above proposition. If the muscle synergies extracted from any one group can explain the same, or even more, amount of EMG variance of another group, then either the two groups are generated by the exact same underlying set of synergies, or some of the synergies of the former group can be linearly combined (i.e., merged) to become synergies of the latter. This generalizability assessment was accomplished first by fitting, using NMF, the synergies of each subject of one group to the EMGs of each subject in every other group, and then, as a benchmark for comparison, fitting the synergies of each subject in the first

group to the data of other subjects in the same group. For both fits, an average R^2 was obtained across subject pairs. The difference between the across-group and within-group cross-fit R^2 values indicates the extent to which the synergies of one group may better describe the EMGs of another, relative to the EMGs of its own group; the more negative the R^2 difference, the less able can the synergies of one group generalize to the other group being fit.

As shown in Supplementary Figure 2, muscle synergies from the less experienced adult runners – including Sedent0/2, and Novice0 – were the most generalizable in describing the EMGs of other groups. Specifically, the Sedent0 synergies, when fit to every other group, produced R^2 values not smaller than those from fits within Sedent0 (Supplementary Figure 2, red solid line; $p>0.05$, 1-tailed t-test). On the other hand, synergies from the more experienced Novice3/6, Exp, and Elite could generalize to the other more experienced groups, and to some extent Presch, but not Sedent0/2 and Novice0. Similarly, the Presch synergies could describe the EMGs of the experienced adults relatively better than those of the less experienced adults. These observations, together with result from our synergy-vector sparseness, merging, and fractionation analyses described in the main text (Fig. 2-5), are consistent with the model that specific Presch muscle synergies are fractionated during development to produce the Sedent0 synergies, some of which in turn are merged over the training course to produce the Exp and Elite synergies. The fact that the Presch synergies could generalize to some extent to the Exp and Elite EMGs (and *vice versa*) suggests that training-induced merging partially, but certainly not completely, reversed developmental fractionation. Indeed, among the 10 most frequently observed merging combinations in Elite (Supplementary Table 3), 3 could be identified as fractionation patterns that derived the Sedent0 synergies from the Presch (Supplementary Table 2), but the other 7 were partially or completely different (see Supplementary Figure 7).

Supplementary Note 3. Elite runners ran with higher energetic efficiency

To quantify the running performance of the different adult groups, we derived a measure of running efficiency based on the relationship between the preferred, body-height-normalized speed of running and the energy loss per kg body weight (vertical direction, over 30 minutes), which in turn was calculated from the vertical ground reaction force (Fig. 1C). Within the data from the untrained (Sedent0/2) and Exp runners, we noticed a positive, statistically significant correlation (r = 0.443, p = 0.0125, 2-tailed t-test) between the preferred running speed (set by treadmill speed) and the 30-minute energy loss (Supplementary Figure 3A, black line). For the other subject groups (Novice and Elite), their energy loss values tended to lie below the regression line that described the data of sedentary and Exp adults (Supplementary Figure 3A). We therefore inferred that each subject's running efficiency (in kJ kg^{-1}) may be approximated by how much the energy loss value lies vertically below the regression line, which denotes the expected energy loss for the less well-performed runners given a certain preferred speed. In other words, per our definition, a positive efficiency value means by how much the energy loss value is smaller than that expected based on baseline data.

We proceeded to compare the running efficiency of the adult groups. Overall, the more trained the subject group (except Exp), the higher the efficiency tended to be (Supplementary Figure 3B). The average efficiency values of the Novice time points were significantly higher than those of Sedent0 and Exp, and the efficiency of Elite, noticeably higher than the rest (Supplementary Figure 3B). Somewhat surprisingly, the efficiency of Exp was much lower than anticipated – in fact lower than the Novice averages and not different from the Sedent0/2 averages.

Perhaps this reflects the diverse, less-than-formal training histories of the Exp subjects despite their experience, which may result in their inadvertent acquisition of certain efficiency-reducing motor patterns (Fig. 5C). But the general trend of an increasing efficiency from Sedent, Novice, to Elite suggests that training can lead to better running performance in the sense of improving the energetic efficiency of running.

We note also that among the adults, running efficiency did not covary with age (p=0.64, 2-tailed t-test for correlation) nor body weight (p=0.37). They correlated weakly with body height (*r*=0.27, p=0.011) because the Elite subjects were taller on average; among the Sedent, Novice and Exp subjects combined, there was no correlation with height (*r*=-0.037, p=0.75).

Supplementary Note 4. Major synergy merging combinations in elite runners

In addition to the 5 energetically relevant merging patterns described in the main text (Fig. 4-5), there were other training-related merging patterns whose presence or absence did not correlate with higher running efficiency. Among the 10 most prevalent merging patterns in Elite, 5 showed a significant increase in prevalence from Sedent0 to Elite (Supplementary Figure 4), and 3 of the 5 were identical to the efficiency-increasing merging combinations described above (*S0*-5+6+8, 5+6+12, 7+11). But whether the other 2 (*S0*- 5+6, 5+11) are relevant to other aspects of running biomechanics related to training remains to be determined.

Supplementary Note 5. Reappearance of some preschooler muscle synergies in the elites

As shown previously in Supplementary Figure 2, the Presch muscle synergies could generalize better to the EMGs of the more experienced adult runners (Novice6, Exp, Elite) than to the less experienced (Sedent0/2, Novice0), suggesting that developmental synergy fractionation may be partially reversed by training-induced synergy merging. Another possibility is that some Presch synergies disappear over the course of development, only to reappear again in Exp and Elite without being involved in any fractionation or merging processes. In fact, one Presch synergy cluster that could not be matched to any Sedent0 cluster, *P*-7 (Fig. 3A, with muscles TA, RF, and RA), reappeared as a subject-specific synergy cluster in Elite that had no apparent counterpart in Sedent0 (Supplementary Figure 1A, cluster *E*-9). To systematically isolate any such synergy, we compiled the muscle synergies of all subjects from all 8 conditions together, and performed *k*-means clustering on them. Any Presch synergy patterns that reappeared in Elite would be indicated by specific clusters prevalent in Presch and Elite but not in the other groups.

In this clustering, a gap statistic measure indicated the presence of 17 clusters. Among them, only 3 (Supplementary Figure 7A, clusters 9, 11, 16) had their two highest frequencies of occurrence observed in Presch and Elite, respectively (Supplementary Figure 7B). For each of these 3 clusters, we then computed averages of the Presch and Elite vectors in the cluster and compared them (Supplementary Figure 7C). Cluster 16, with muscles TA, RF, and RA in both Presch and Elite, corresponded well to the unmatched clusters *P*-7 (Fig. 3A) and *E*-9 (Supplementary Figure 1A) identified earlier. The other 2 reappearing clusters appeared to indicate how fractionation was reversed by merging. Cluster 9, with muscles TA and HAM (*cf. P*-4 in Fig. 3A and *E*-5 in Supplementary Figure 1A) can be explained by combining *S0*-4 (HAM) and *S0*-7 (TA). For cluster 11, the Presch (*cf. P*-6 in Fig. 3A) and Elite (*cf. E*-8 in Supplementary Figure 1A) averages differed in that the Presch vector had more activations in TA, but the Elite, more in the ankle extensors MGN and LGN. This difference can be readily accounted for by noting that

the Presch vector was fractionated into *S0*-6, 7 and 8 in Sedent0 (Fig. 3B, 3C), but the Elite vector was instead formed by merging *S0*-5, 6, and 8 without *S0*-7 (Fig. 4A). This partial, but not complete, reversal of fractionation during merging, as suggested by cluster 11, was the dominant factor in explaining the apparent return of Presch patterns in Elite given the high prevalence of cluster 11 in both Presch and Elite, and that its frequency increased gradually over the training stages (Supplementary Figure 7B, red).

Supplementary Note 6. Comparison with data reported in Yokoyama *et al.* (2016)

In a recent paper, Yokoyama *et al.* (2016) (*1*) ask whether humans employ distinct sets of muscle synergies to accomplish walking and running at different speeds. Since muscle synergies from both non-runners and runners at different running speeds were presented by these authors, it should be instructive to compare their results with ours even though in their work, synergies from the two groups were not explicitly compared in detail. We assume that their runners (age 20-24; 5-11 years of training) had similar levels of experience with our Elite group (age 27-46; 3-30 years of training, mean of 8.97 years) despite age difference. Also, the self-selected preferred running speeds of our Sedentary group (6.2 ± 0.9 km h^{-1}) likely correspond to the slow running (SR) speeds of their non-runners (8.6-9.7 km h^{-1}), and that of our Elite group (12 km h^{-1}), to the moderate running (MR) speeds of their runners (12.6-13.3 km h^{-1}). Thus, we will attempt to compare the non-runners' SR synergies and the runners' MR synergies in Yokoyama *et al.* with our results.

A close examination of the synergies of the two groups presented in Fig. 6 and Table 1 of Yokoyama *et al.* (*1*) reveals that our E2 efficiency-enhancing synergy merging combination (*S0*-5+6+8/5+6+12) appears to be present only in their runners but not non-runners. This combination, comprising activities in the ankle plantar-flexors, knee extensors, TFL and GLUT, is most similar to their synergy M5 (RF, VL, VM, GLUT, TFL, and smaller components in LGN and SOL), which is represented only in their runners (MR, 6 of 8 subjects) but not non-runners (SR, 0 of 8). This is consistent with our observation that E2 was prevalent in the Elite but not Sedentary groups (Fig. 4C).

Likewise, there are signs that our two efficiency-reducing synergy combinations, R1 and R2, were present in the non-runners but not the runners in Yokoyama *et al.* Our combination R1 (*S0*-3+12) includes LAT DOR (not recorded in Yokoyama *et al.*), ES, GLUT, and TFL; in the synergy cluster averages of their M7, ES and GLUT are prominent only in non-runners but not runners. Also, our combination R2 (*S0*-4+5+7) includes TA, the plantar-flexors and HAM; in their M3, while the average for non-runners shows activities in the plantar-flexors and biceps femoris (part of HAM), the average for runners shows only the plantar-flexors without HAM.

The above comparisons, though qualitative in nature, nonetheless argue for the consistency of our results with those of Yokoyama *et al.*, thus further supporting the validity of our findings.

SUPPLEMENTARY FIGURES

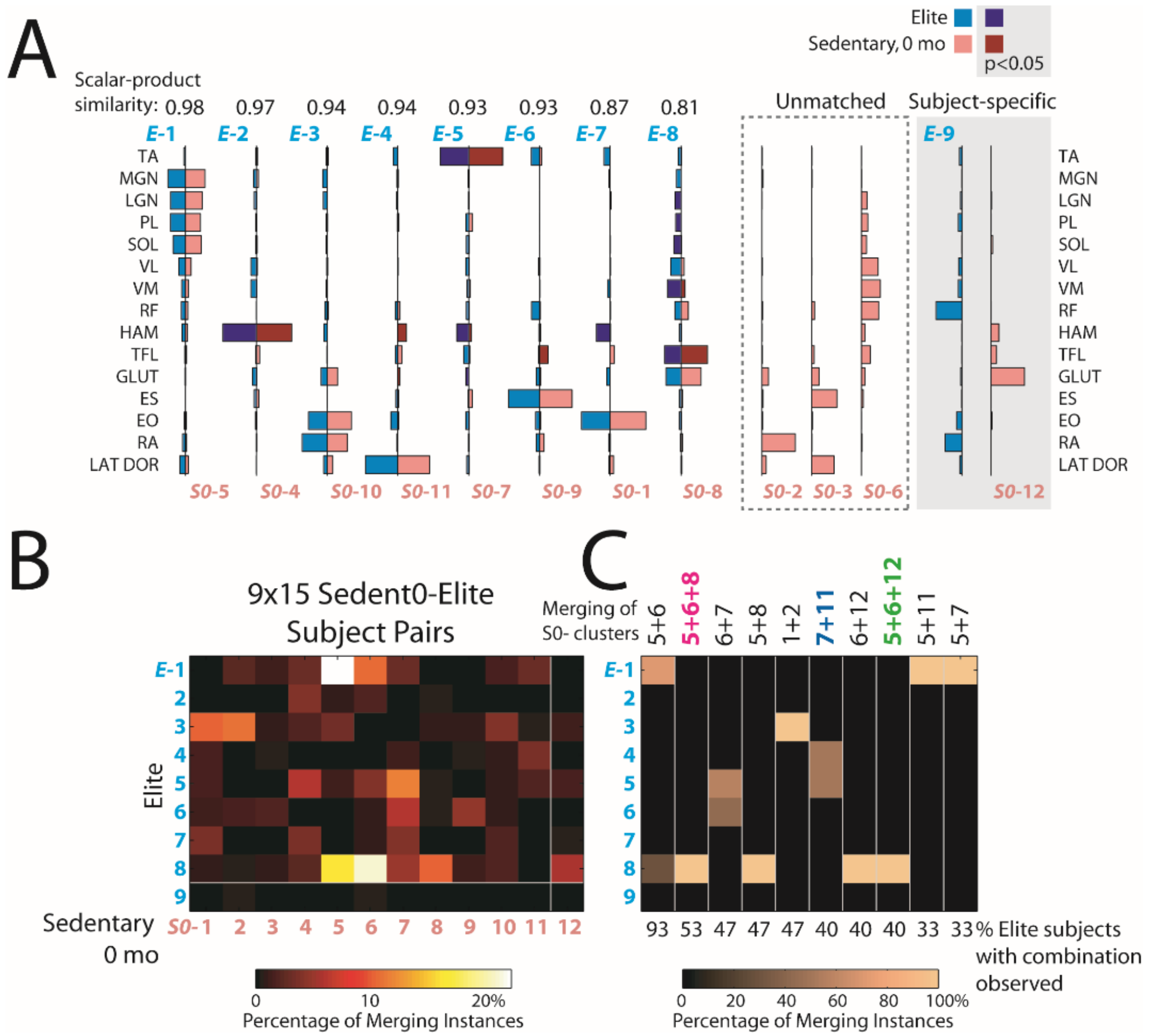

Supplementary Figure 1. Explaining Elite synergies by merging Sedent0 synergies. (**A**) Shown are the *k*-means muscle-synergy cluster centroids of Elite (blue, *E*-1 to 9; numbers of synergies in clusters 1 to 9 were, n = 15, 5, 9, 12, 9, 12, 5, 16, 4) and Sedent0 (pink, *S0*-1 to 12; n = 5, 5, 4, 5, 9, 8, 9, 3, 4, 4, 3, 2), matched by maximizing scalar product (SP). After matching, the component values of every muscle of each cluster were compared between the 2 groups (dark blue and red, p<0.05; 2-tailed t-test). The subject-specific clusters were excluded from matching. In all matched pairs, the Elite synergy centroid tended to have active components over more muscles (less sparse) then its Sedent0 counterpart (except pair of *E*-4/*S0*-11). The *S0*- labelling here matches that in Fig. 3-4 and Supplementary Table 1-3. P value for cluster 2: HAM, 0.011. P values for cluster 5: TA, 0.028; HAM, 0.046. P value for cluster 7: HAM, 0.043. P values for cluster 8: LGN, 0.047; PL, 0.048; SOL, 0.047; VM, 0.040; TFL, 0.0076. (**B**) Synergy merging patterns were further characterized by comparing the synergy sets of every Sedent0/Elite subject pairs (9 x 15 = 135 pairs). Shown is a heat map depicting the percentage of all detected merging instances that involved merging a synergy from any Sedent0 (*S0*-) cluster to become a synergy in any Elite (*E*-) cluster. Most instances involved merging *S0*-1 and 2 into *E*-3 and *S0*-5, 6, and/or 8 into *E*-1 or 8.

Grey horizontal and vertical lines separate the matched/unmatched from the subject-specific clusters for Elite and Sedent0, respectively, as indicated in (A). (**C**) We list the 10 most prevalent merging combinations in Elite (% subjects possessing each combination listed below the graph), and for each, we show as a heat map how its instances of merging distribute across the 9 Elite synergy clusters (*E*-) shown in (A). All instances (100%) of 4 combinations (including 2 efficiency-enhancing ones) contribute to synergies in *E*-8 – a cluster with co-activations of hip, knee and ankle extensors, and one that is the least well-matched to Sedent0 clusters in (A). Source data for all panels are available as a Source Data file.

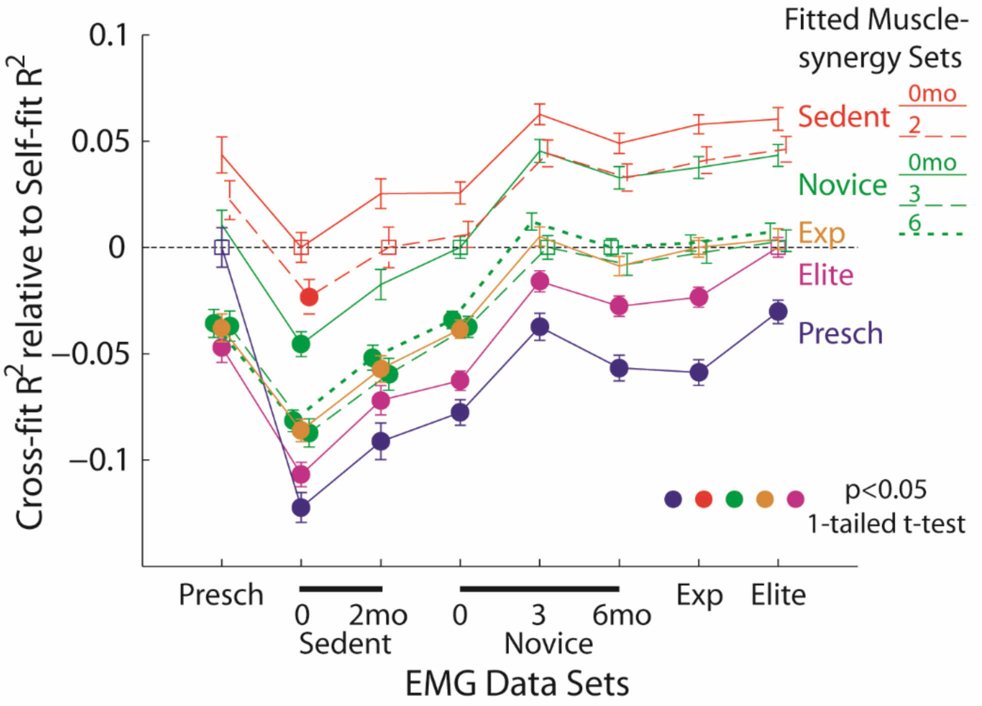

Supplementary Figure 2. Across-group generalizability of the muscle synergies from each group. The muscle synergies of each subject were fitted to the EMGs of other subjects from other groups and in the subject's own group. For any two groups ("A" and "B"), generalizability of the synergies extracted from "A" to EMGs of "B" is then indicated by the A-to-B cross-fit R^2 subtracted by the within-A cross-fit R^2. In this figure, the R^2-difference values (mean ± SE) obtained from the muscle synergies of the same conditions are connected by solid or dotted lines of different colors (legend on right). For each line, the average difference value at the line's condition (□) is by definition zero, and whether the values at other conditions are significantly smaller than that at the line's condition are assessed by a 1-tailed t-test (●, $p<0.05$). As can be seen, the muscle synergies from Sedent0/2 (red lines) and Novice0 (solid green line) generalized to almost all other conditions (with difference in cross-fit $R^2 > 0$). As examples, we list the P values obtained for 3 of the 8 synergy sets. P values for the Presch synergies (for fits to Presch to Elite EMGs): p = Nil, $1\text{x}10^{-20}$, $8\text{x}10^{-12}$, $1\text{x}10^{-12}$, $3\text{x}10^{-4}$, $1\text{x}10^{-7}$, $4\text{x}10^{-8}$, 0.0015. P values for Novice6: p = $1\text{x}10^{-6}$, $5\text{x}10^{-30}$, $4\text{x}10^{-13}$, $4\text{x}10^{-9}$, 0.983, Nil, 0.644, 0.894. P values for Sedent0: p = 0.999, Nil, 0.994, 0.998, 1, 1, 1, 1. Sample sizes for the 5 groups were, n = 10 (Presch), 9 (Sedent), 14 (Novice), 15 (Exp), 15 (Elite). Source data for all panels are available as a Source Data file.

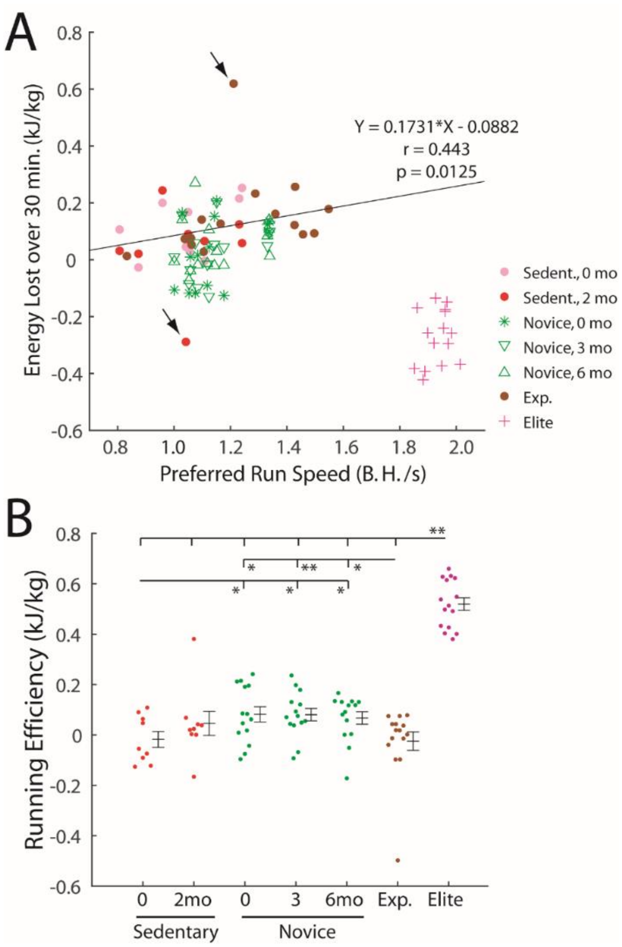

Supplementary Figure 3. Quantifying energetic efficiency of running. (A) Running efficiency was calculated based on the relationship between the subject's preferred, body-height-normalized running speed, and the energy loss per kg body weight (vertical direction, over 30 minutes) derived from the vertical ground reaction force. A linear regression was performed over the data from the Sedent0/2 and Exp runners (black line; r=0.443, p=0.0125, 2-tailed t-test) with two obvious outliers omitted from regression (black arrows). Each subject's running efficiency (in kJ kg^{-1}) was approximated by noting how much the subject's energy loss value lies vertically below the regression line, which denotes the energy loss expected for less well-performed runners at a certain speed. (**B**) Running efficiency of the 7 conditions from the 4 subject groups (mean ± SE). Elite had significantly higher efficiency than the rest (p=6.6×10^{-10}, Kruskal-Wallis over data of 4 subject groups; post hoc multiple comparison at α=0.05); Novice in turn had higher efficiency than Exp (at α=0.058), and marginally higher efficiency than Sedent (at α=0.13). Additional tests were also performed between pairs of conditions (*, p<0.05; **, p<0.01; 2-tailed t-test or Mann-Whitney). P values for comparisons involving Sedent0: p = 0.0407 (Novice0), 0.0214 (Novice3), 0.0455 (Novice6). P values for comparisons involving Exp: p = 0.0424 (Novice0), 0.0064 (Novice3), 0.0107 (Novice6). P values for comparisons involving Elite: p = 5×10^{-12} (Sedent0), 8×10^{-5} (Sedent2), 1×10^{-11} (Novice0), 8×10^{-13} (Novice3), 5×10^{-13} (Novice6), 3×10^{-6} (Exp). Sample sizes for the 4 groups were, n = 9 (Sedent), 14 (Novice), 15 (Exp), 15 (Elite). Source data for all panels are available as a Source Data file.

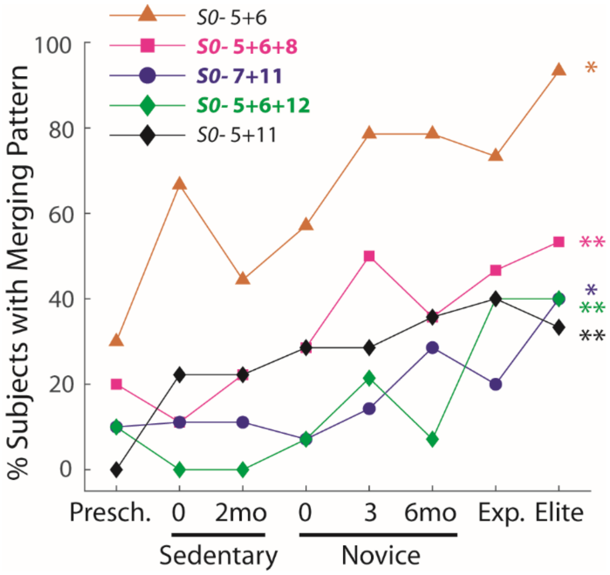

Supplementary Figure 4. Major merging combinations in Elite with increases in prevalence across groups. Among the 10 most prevalent synergy merging combinations in Elite, 5 of them (patterns 1, 2, 6, 8, and 9 in Supplementary Table 3) showed a statistically significant increase in prevalence from the least to most experienced adult groups. Of these 5, 3 of them (patterns 2, 6, 8 in Supplementary Table 3) were identified to be efficiency-enhancing merging combinations among adult runners (see main text). We do not know whether the remaining 2 have any biomechanical significance. The Pearson's *r* (from Sedent0 to Elite) and its associated p value (*, $p<0.05$; **, $p<0.01$; 2-tailed t-test) for the above combinations are: *S0*-5+6, $r=0.77$, $p=0.043$; 5+6+8, $r=0.90$, $p=0.0058$; 7+11, $r=0.83$, $p=0.021$; 5+6+12, $r=0.88$, $p=0.0091$; 5+11, $r=0.88$, $p=0.0096$. Source data for all panels are available as a Source Data file.

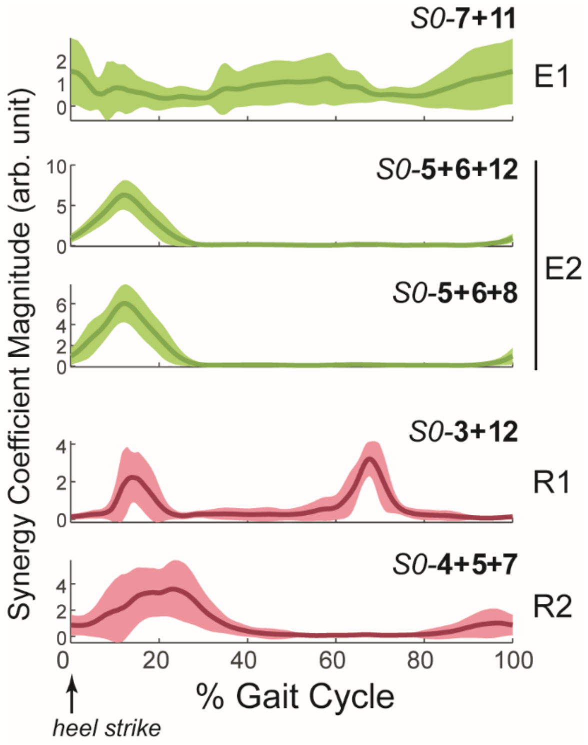

Supplementary Figure 5. Average temporal coefficients of the biomechanically relevant synergy merging combinations. For each combination, all adults possessing the combination were considered. Each running cycle, with on- and offset defined by moments of heel strike, was resampled to 1000 time points before averaging. Combinations *S0*-5+6+12 and 5+6+8 were active in stance, and may function to increase efficiency by enhancing propulsion through stabilization of hip extension; *S0*-4+5+7, also active in stance, may reduce efficiency by increasing ankle stiffness and vertical loading rate. *S0*-7+11 and 3+12 were active through the cycle, and may respectively increase or decrease efficiency through their influences on the coordination between arm- and leg-swing. See Discussion. Numbers of subjects included, from top to bottom, were, n = 19, 18, 36, 9, 14. Cycle averages (across subjects) ± SD are shown. Green, efficiency-enhancing merging combinations (E1 and E2); red, efficiency-reducing merging combinations (R1 and R2). Source data for all panels are available as a Source Data file.

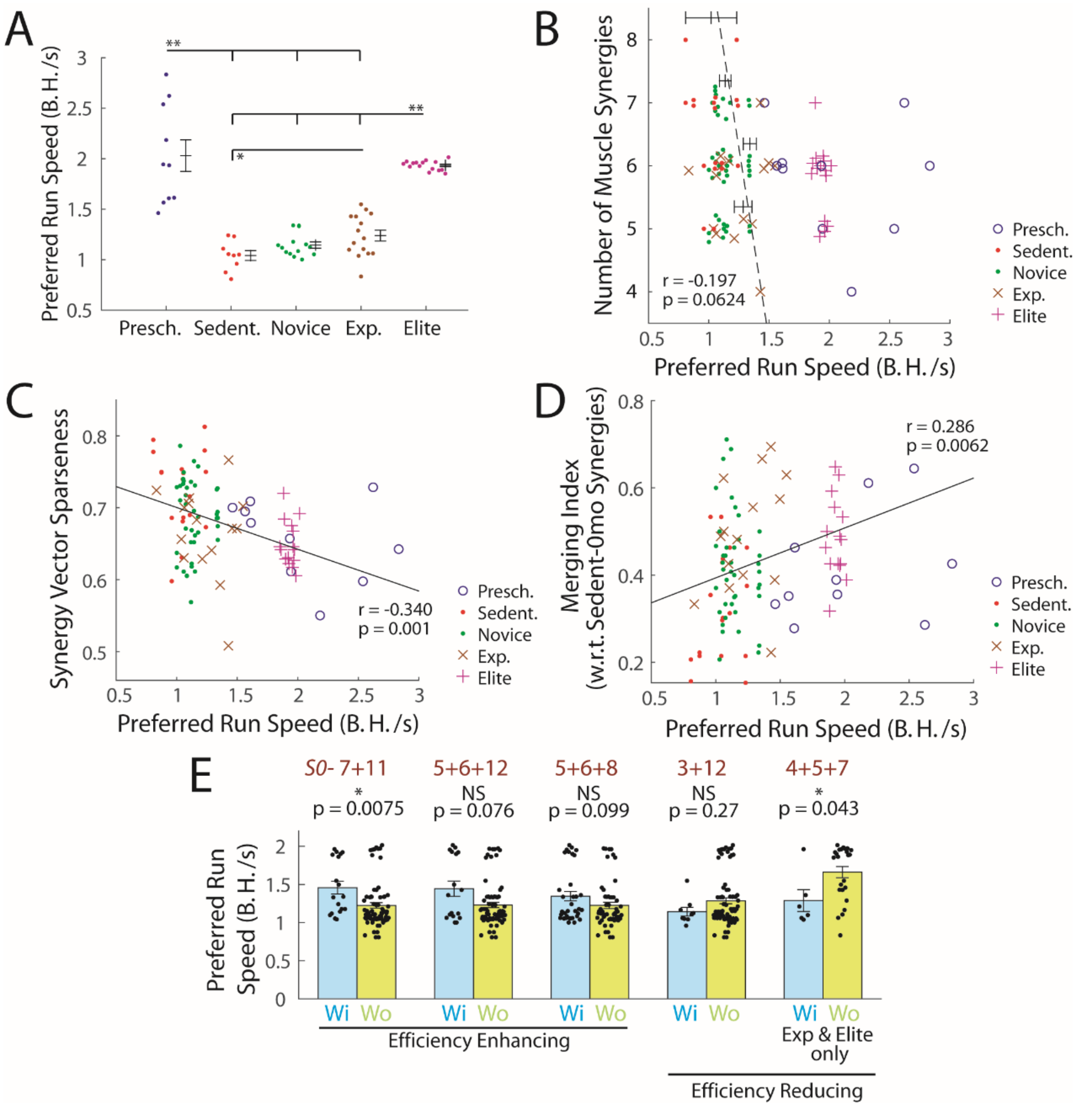

Supplementary Figure 6. Correlations of indicators of muscle-synergy change with preferred running speed. (A) The preferred body-height (B. H.)-normalized running speed of the subject groups (mean ± SE). Among adults, as subjects gained training experience, the preferred speed also increased. Speeds of Presch and Elite were noticeably higher than the rest; the Exp speed was also significantly higher than that of Sedent ($p=1.4\times10^{-17}$, ANOVA with multiple comparison). Additional tests between pairs of groups were performed (*, $p<0.05$; *, $p<0.01$; 2-tailed t-test). P values for comparisons involving Presch: $p = 2\times10^{-5}$ (Sedent), 1×10^{-6} (Novice), 1×10^{-5} (Exp). P values for comparison involving Sedent, p = 0.0209 (Exp). P values for comparisons involving Elite, $p = 2\times10^{-16}$ (Sedent), 5×10^{-20} (Novice), 5×10^{-13} (Exp). Sample sizes for the 5 groups were, n = 10 (Presch), 9 (Sedent), 14 (Novice), 15 (Exp), 15 (Elite). **(B)-(D)** Correlations of the three indicators of across-group muscle-synergy changes discussed in main text (Fig. 2) with the preferred running speed. The indicators are the number of muscle synergies, or dimensionality (in (B), Fig. 2A), vector sparseness (in (C), Fig. 2B), and the Merging Index (in (D), Fig. 2C). The regression line and its associated *r* and p values (2-tailed t-test) shown on figure were obtained by regressing only the adult data without Presch. When both Presch and adults were considered, the

values were: dimensionality, *r*=-0.21, p=0.039, 2-tailed t-test; vector sparseness, *r*=-0.36, p=2.4x10^{-4}; Merging Index, *r*=0.23, p=0.021. All correlations are significant or nearly significant, but the |*r*|'s are low. See Discussion. **(E)** Comparisons of the preferred running speeds of the subjects with (Wi, the 'have's', cyan bars) or without (Wo, the 'have-not's', green bars) each of the 5 biomechanically relevant merging combinations, respectively (mean ± SE; numbers of Wi subjects, from left to right, n = 18, 17, 34, 9, 6; numbers of Wo subjects, n = 72, 73, 56, 81, 24). For *S0*-4+5+7, following Fig. 5B, only Exp and Elite subjects were considered. Of the 5 combinations, the have's and have-not's of 3 combinations had speeds that were not significantly different (NS, p>0.05; 2-tailed Mann-Whitney), thus further arguing that the combinations we identified are functionally more related to training-induced increase in running efficiency than to increase in running speed. Source data for all panels are available as a Source Data file.

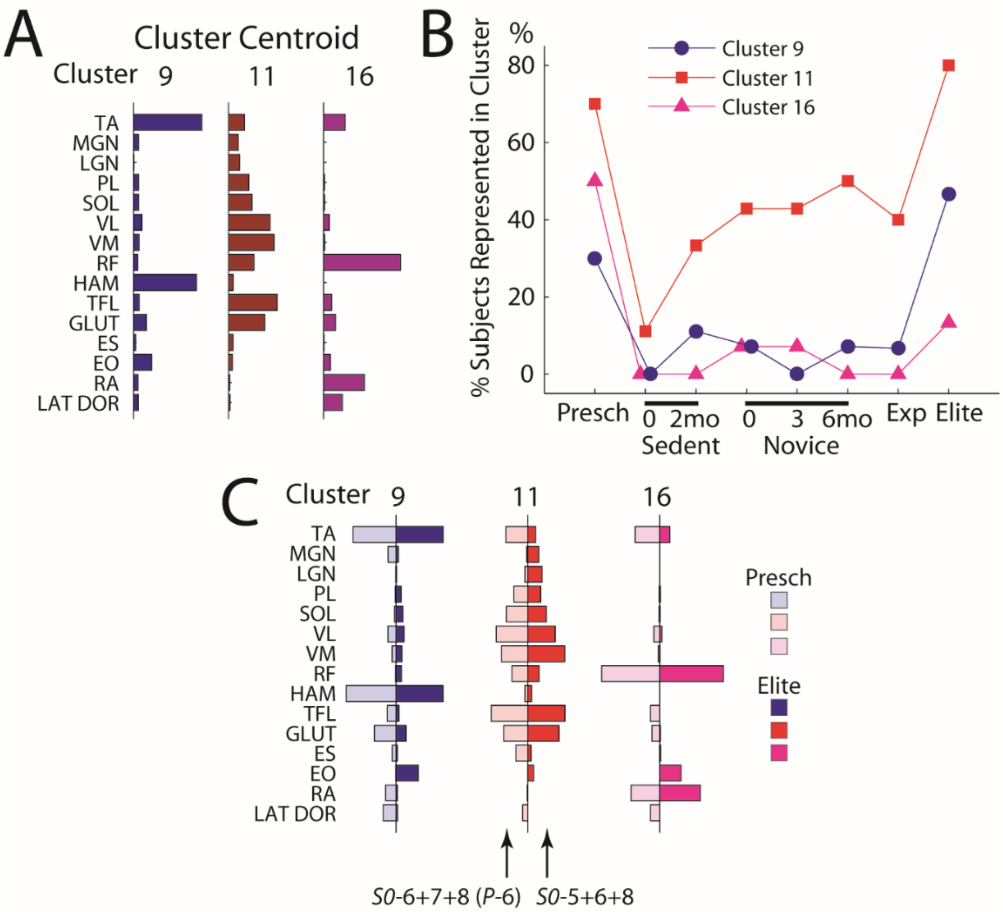

Supplementary Figure 7. Reappearance of preschooler synergy patterns in elite runners. (**A**) To assess whether synergy fractionation patterns associated with motor development is reversed by training-induced synergy merging, we performed *k*-means clustering on the set of muscle synergies compiled from all subjects from all 8 conditions. Of the 17 clusters identified, 3 (clusters 9, 11, 16; their cluster centroids shown here) had their two highest frequencies of occurrence observed in Presch and Elite, respectively. The numbers of synergies grouped into the clusters were, n = 14 (cluster 9), 48 (cluster 11), and 9 (cluster 16). (**B**) Frequencies of occurrence (% subjects) of the 3 clusters shown in (A), across all 8 conditions (blue, cluster 9; red, cluster 11; magenta, cluster 16). (**C**) For each of the 3 clusters in (B), we computed averages of the Presch (lighter colors) and Elite (darker colors) vectors in the cluster and compared them. Note that the Presch and Elite averages are noticeably different in cluster 11, because the Presch vector would be fractionated into *S0*-6, 7 and 8 in Sedent0, but the Elite vector was instead formed by merging *S0*-5, 6, and 8 without *S0*-7. This partial, but not complete, reversal of fractionation during merging partially explains the apparent return of Presch patterns in Elite. See text for details. Numbers of Presch synergies in clusters 9, 11, and 16 were, n = 3, 7, 5; numbers of Elite synergies, n = 7, 12, 2. Source data for all panels are available as a Source Data file.

SUPPLEMENTARY TABLES

Supplementary Table 1. Muscles represented in the muscle synergies of the sedentary adults at 0 month.

Synergy cluster	Muscles
***S0*-1**	(TFL), **EO**, (LAT DOR)
***S0*-2**	**RA**, (GLUT), (LAT DOR)
***S0*-3**	(GLUT), **ES**, **LAT DOR**
***S0*-4**	**HAM**, (TFL)
***S0*-5**	**MGN**, **LGN**, **PL**, **SOL**, (VL)
***S0*-6**	(LGN), (PL), (SOL), **VL**, **VM**, **RF**, (HAM), **TFL**, (GLUT)
***S0*-7**	**TA**, (PL), (ES)
***S0*-8**	(VL), (VM), (RF), **TFL**, **GLUT**
***S0*-9**	(TFL), **ES**, (RA)
***S0*-10**	**GLUT**, **EO**, **RA**, (LAT DOR)
***S0*-11**	(HAM), (TFL), **LAT DOR**
***S0*-12**	(HAM), (TFL), **GLUT**

The 12 *S0*- clusters here are as shown in Fig. 3 and Supplementary Figure 1. Only the muscles with component values ≥ cos(85°) = 0.0872 after normalization of the muscle-synergy vector magnitude are listed. Major muscles of each cluster with components ≥ $1/\sqrt{15}$ = 0.2582 are shown in bold, otherwise in parentheses. Abbreviations: LAT DOR, latissimus dorsi; EO, external oblique; RA, rectus abdominalis; ES, erector spinae; GLUT, gluteus maximus; TFL, tensor fasciae latae; VM, vastus medialis; VL, vastus lateralis; RF, rectus femoris; HAM, hamstring, or bicep femoris; TA, tibialis anterior; MGN, medial gastrocnemius; LGN, lateral gastrocnemius; SOL, soleus; PL, peroneus longus.

Supplementary Table 2. Major patterns of muscle synergy fractionation associated with child-to-adult development.

Pattern	Fractionations produced in sedentary group (*S0*)	Percentage of preschoolers showing fractionation (%)
1	*S0*-**5**, *S0*-**7**	70
2	*S0*-**9**, *S0*-**11**	60
3	*S0*-**6**, *S0*-**7**	50
4	*S0*-**6**, *S0*-**7**, *S0*-**9**	40
5	*S0*-**6**, *S0*-**7**, *S0*-**8**	40
6	*S0*-**2**, *S0*-**6**, *S0*-**7**	40
7	*S0*-**7**, *S0*-**8**	30
8	*S0*-**6**, *S0*-**8**	30
9	*S0*-**6**, *S0*-**7**, *S0*-**12**	30
10	*S0*-**5**, *S0*-**7**, *S0*-**8**	30
11	*S0*-**5**, *S0*-**6**	30
12	*S0*-**4**, *S0*-**10**	30
13	*S0*-**2**, *S0*-**9**	30
14	*S0*-**2**, *S0*-**4**	30
15	*S0*-**2**, *S0*-**3**	30

These patterns were revealed by comparing the synergies of each preschooler with those of each sedentary adult at 0 month (10 x 9 = 90 subject pairs). The *S0*- clusters listed are as shown in Fig. 3 and Supplementary Figure 1. The muscles activated in each cluster are shown in Supplementary Table 1.

Supplementary Table 3. Major patterns of muscle synergy merging associated with training on running.

Pattern	Merging pattern observed in elite	Percentage of elite with merging pattern (%)
1	*S0*-**5**, *S0*-**6**	93
2	*S0*-**5**, *S0*-**6**, *S0*-**8**	53
3	*S0*-**6**, *S0*-**7**	47
4	*S0*-**5**, *S0*-**8**	47
5	*S0*-**1**, *S0*-**2**	47
6	*S0*-**7**, *S0*-**11**	40
7	*S0*-**6**, *S0*-**12**	40
8	*S0*-**5**, *S0*-**6**, *S0*-**12**	40
9	*S0*-**5**, *S0*-**11**	33
10	*S0*-**5**, *S0*-**7**	33

These patterns were revealed by comparing the synergies of each sedentary adult at 0 month with those of each elite (9 x 15 = 135 subject pairs). The *S0*- clusters listed are as shown in Fig. 3 and Supplementary Figure 1. The muscles activated in each cluster are shown in Supplementary Table 1.

SUPPLEMENTARY REFERENCE

1. H. Yokoyama, T. Ogawa, N. Kawashima, M. Shinya, K. Nakazawa, Distinct sets of locomotor modules control the speed and modes of human locomotion, *Sci. Rep.* **6**, 36275 (2016).
