# Supplementary material for: Plasticity of muscle synergies through fractionation and merging during development and training of human runners
Source: Nat Commun. 2020 Aug 31;11:4356. doi: 10.1038/s41467-020-18210-4 (PMC7459346; doi:10.1038/s41467-020-18210-4)
Supplement: Supplementary file 3 — Reporting Summary [file 41467_2020_18210_MOESM3_ESM.pdf]

nature research

Corresponding author(s): Vincent C. K. Cheung; Roy T. H. Cheung

Last updated by author(s): Jun 20, 2020

Reporting Summary

Nature Research wishes to improve the reproducibility of the work that we publish. This form provides structure for consistency and transparency in reporting. For further information on Nature Research policies, see our Editorial Policies and the Editorial Policy Checklist.

Statistics

For all statistical analyses, confirm that the following items are present in the figure legend, table legend, main text, or Methods section.

n/a	Confirmed	
☐	☒	The exact sample size (*n*) for each experimental group/condition, given as a discrete number and unit of measurement
☐	☒	A statement on whether measurements were taken from distinct samples or whether the same sample was measured repeatedly
☐	☒	The statistical test(s) used AND whether they are one- or two-sided<br>*Only common tests should be described solely by name; describe more complex techniques in the Methods section.*
☐	☒	A description of all covariates tested
☐	☒	A description of any assumptions or corrections, such as tests of normality and adjustment for multiple comparisons
☐	☒	A full description of the statistical parameters including central tendency (e.g. means) or other basic estimates (e.g. regression coefficient) AND variation (e.g. standard deviation) or associated estimates of uncertainty (e.g. confidence intervals)
☐	☒	For null hypothesis testing, the test statistic (e.g. *F*, *t*, *r*) with confidence intervals, effect sizes, degrees of freedom and *P* value noted<br>*Give P values as exact values whenever suitable.*
☒	☐	For Bayesian analysis, information on the choice of priors and Markov chain Monte Carlo settings
☒	☐	For hierarchical and complex designs, identification of the appropriate level for tests and full reporting of outcomes
☐	☒	Estimates of effect sizes (e.g. Cohen's *d*, Pearson's *r*), indicating how they were calculated

Our web collection on statistics for biologists contains articles on many of the points above.

Software and code

Policy information about availability of computer code

Data collection	All data reported in this MS (EMGs, kinematics, and ground reaction forces) were collected using commercially available equipment controlled by data acquisition software designed by the equipment manufacturer. We have not relied on any custom software for data collection. EMG data were collected using the software Trigno Control Utility (version 2.6.12, Delsys; Natick, MA, USA). Motion capture and force plate data were collected using the software Vicon Nexus (version 1.8.5 for treadmill trials; version 2.4.0 for overground trials; Vicon; Oxford, UK).
Data analysis	Data analysis was performed on Matlab (version R2016b and R2019b; Mathworks, Natick, MA, USA) using custom code and software. The algorithms employed have all been previously published, and we should be glad to make the Matlab codes available to anyone upon request.

For manuscripts utilizing custom algorithms or software that are central to the research but not yet described in published literature, software must be made available to editors and reviewers. We strongly encourage code deposition in a community repository (e.g. GitHub). See the Nature Research guidelines for submitting code & software for further information.

Data

Policy information about availability of data

All manuscripts must include a data availability statement. This statement should provide the following information, where applicable:

- Accession codes, unique identifiers, or web links for publicly available datasets
- A list of figures that have associated raw data
- A description of any restrictions on data availability

All data that support the findings of this study are available from the corresponding authors upon reasonable request. An example of the raw data collected is shown in Fig. 1A and 1C.
The data that support the findings of this study are available at PhysioNet at https://physionet.org/. The source data underlying Figs. 1-7 and all figures in Supplementary Information are provided as a Source Data file.

Field-specific reporting

Please select the one below that is the best fit for your research. If you are not sure, read the appropriate sections before making your selection.

- [x] Life sciences
- [ ] Behavioural & social sciences
- [ ] Ecological, evolutionary & environmental sciences

For a reference copy of the document with all sections, see nature.com/documents/nr-reporting-summary-flat.pdf

Life sciences study design

All studies must disclose on these points even when the disclosure is negative.

Sample size	We did not perform any specific sample size calculation. But we selected the number of participants based on another well-known study concerning development and muscle synergies (Dominici et al., Science, 334: 997, 2011). In that study, the authors were able to reach sound conclusions by analyzing 10 subjects in each group of preschoolers, toddlers and adults. In ours, we had 10 preschoolers, 9 sedentary adults, 14 novice runners, 15 experienced runners and 15 elites. Thus, our sample size is comparable to - and in fact, for 3 adult groups, larger than - that in Dominici et al.
Data exclusions	We excluded the data of 1 subject in the sedentary adult group, because the subject dropped out after the first session without completing the second session, thus making the data from this subject unusable. Otherwise, no data was excluded from our analysis.
Replication	The analysis was initially performed by one of the co-first authors. Then, the other co-first author succeeded in replicating the results of the analysis. We did not note any incidence of failing to reproduce or replicate the analytic findings from our collected data.
Randomization	Randomization of the participants to the different groups is not relevant to the design of the current study, because group assignment was based on the reported age and prior running experience of each subject.
Blinding	The investigators were not blinded to the group assignment during both data collection and data analysis. For data collection, because different subject groups were recorded with different number of time points, it was impossible to blind the investigators. For data analysis, since one major goal was to correlate the analytic findings with the age and/or prior experience of the runners, again it was impossible for the analysts to be blinded to group allocation.

Reporting for specific materials, systems and methods

We require information from authors about some types of materials, experimental systems and methods used in many studies. Here, indicate whether each material, system or method listed is relevant to your study. If you are not sure if a list item applies to your research, read the appropriate section before selecting a response.

Materials & experimental systems

n/a	Involved in the study	
x		Antibodies
x		Eukaryotic cell lines
x		Palaeontology and archaeology
x		Animals and other organisms
	x	Human research participants
x		Clinical data
x		Dual use research of concern

Methods

n/a	Involved in the study	
x		ChIP-seq
x		Flow cytometry
x		MRI-based neuroimaging

Human research participants

Policy information about studies involving human research participants

Population characteristics	Healthy preschoolers (age = 4.2 ± 1.6) of either gender, and healthy adults of either gender with varying prior experience with running training (sedentary adults, age 30.2 ± 4.8; novice runners, age = 45.3 ± 6.8; experienced, age = 43.0 ± 11.3; elite marathoners, age = 37.3 ± 6.4).
Recruitment	The preschoolers and sedentary adults were recruited through online advertisements, and the adult runners, from various local running clubs. We are confident that the selected runners were not a biased population because they were trained by different coaches from different running clubs.<br>We cannot entirely exclude the presence of any self-selection bias in our cohorts of preschoolers and sedentary subjects, but we expect the chance for such bias to exist - and if so, to impact on our results - is very small. The preschoolers whose parents would respond to our advertisement are unlikely to have had developmental trajectories that are vastly different from those of other average preschoolers, because all preschoolers in this study were healthy individuals without any known developmental or sensorimotor impairment at the time of recording. Similarly, the sedentary adults who would respond to our advertisement are unlikely to have more or less prior physical activities or running experience, because they are by definition sedentary, and must have no ongoing or prior history of running training for them to be included in the study.
Ethics oversight	Departmental Research Committee of the Department of Rehabilitation Sciences, Hong Kong Polytechnic University (protocol number HSEARS20150730002)

Note that full information on the approval of the study protocol must also be provided in the manuscript.
